# 6-Cyclo­hexyl­meth­yl-5-ethyl-2-[(2-oxo-2-phenyl­eth­yl)sulfan­yl]pyrimidin-4(3*H*)-one

**DOI:** 10.1107/S1600536811003175

**Published:** 2011-01-29

**Authors:** Wan-Lu Yan, Qiong Guo, Cong Li, Xiao-Ying Ji, Yan-Ping He

**Affiliations:** a School of Chemical Science and Technology, Key Laboratory of Medicinal Chemistry for Natural Resources (Ministry of Education), Yunnan University, Kunming 650091, People’s Republic of China; b Laboratory of Molecular Medicine, College of Pharmaceutical Science, Soochow University, Suzhou 215123, People’s Republic of China

## Abstract

In the title compound, C_21_H_26_N_2_O_2_S, the cyclo­hexane ring adopts a chair conformation. The angle at the methyl­ene bridge linking the pyrimidine and cyclo­hexane rings is 113.41 (13)°. This is in the range considered optimal for maximum activity of non-nucleoside reverse transcriptase inhibitors. In the crystal, mol­ecules are connected into centrosymmetric dimers *via* pairs of N—H⋯O hydrogen bonds.

## Related literature

For the biological activity of 2-alkyl­sulfanyl-6-benzyl-3,4-dihydro­pyrimidin-4(3*H*)-one derivatives, which show remarkable anti-HIV-1 activity, see: He *et al.* (2011 ▶); Ettorre *et al.* (1996 ▶). For related structures, see: Ettorre *et al.* (1998 ▶); Rao *et al.* (2007 ▶); Zhang *et al.* (2008 ▶).
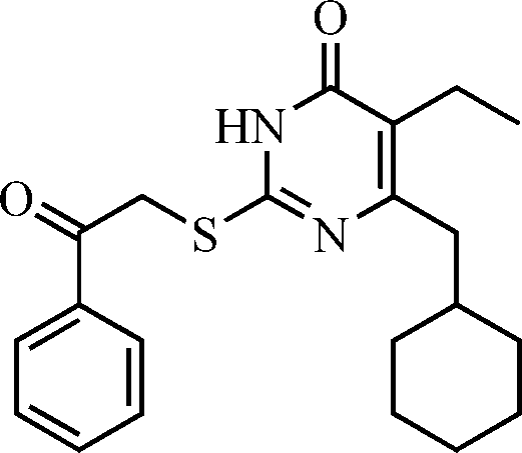


## Experimental

### 

#### Crystal data

C_21_H_26_N_2_O_2_S
*M* *_r_* = 370.50
Triclinic, 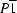

*a* = 7.516 (5) Å
*b* = 10.960 (5) Å
*c* = 12.490 (5) Å
α = 84.082 (5)°
β = 78.925 (5)°
γ = 80.267 (5)°
*V* = 992.5 (9) Å^3^
*Z* = 2
Mo *K*α radiation
μ = 0.18 mm^−1^
*T* = 293 K
0.46 × 0.23 × 0.17 mm

#### Data collection

Bruker SMART CCD area-detector diffractometer
Absorption correction: multi-scan (*SADABS*; Bruker, 2004 ▶) *T*_min_ = 0.944, *T*_max_ = 0.975
7097 measured reflections
4158 independent reflections
3203 reflections with *I* > 2σ(*I*)
*R*_int_ = 0.019

#### Refinement

*R*[*F* ^2^ > 2σ(*F* ^2^)] = 0.040
*wR*(*F* ^2^) = 0.129
*S* = 1.03
4158 reflections
237 parameters
H-atom parameters constrained
Δρ_max_ = 0.18 e Å^−3^
Δρ_min_ = −0.18 e Å^−3^

### 

Data collection: *SMART* (Bruker, 2004 ▶); cell refinement: *SAINT* (Bruker, 2004 ▶); data reduction: *SAINT*; program(s) used to solve structure: *SHELXS97* (Sheldrick, 2008 ▶); program(s) used to refine structure: *SHELXL97* (Sheldrick, 2008 ▶); molecular graphics: *SHELXTL* (Sheldrick, 2008 ▶); software used to prepare material for publication: *SHELXTL*.

## Supplementary Material

Crystal structure: contains datablocks I, global. DOI: 10.1107/S1600536811003175/tk2710sup1.cif

Structure factors: contains datablocks I. DOI: 10.1107/S1600536811003175/tk2710Isup2.hkl

Additional supplementary materials:  crystallographic information; 3D view; checkCIF report

## Figures and Tables

**Table 1:** Hydrogen-bond geometry (Å, °)

*D*—H⋯*A*	*D*—H	H⋯*A*	*D*⋯*A*	*D*—H⋯*A*
N2—H2*A*⋯O2^i^	0.86	1.90	2.743 (2)	168

Symmetry code: (i) 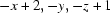.

**Fig. 1.**
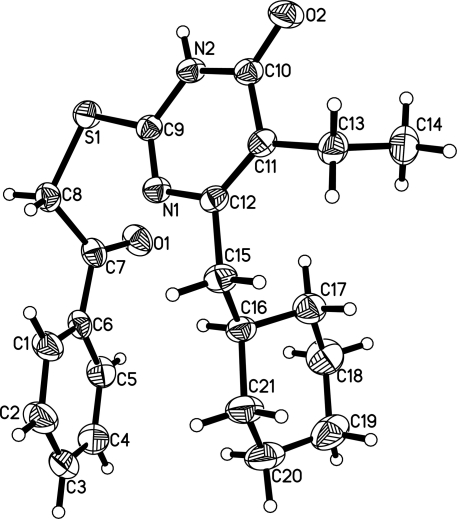
The molecular structure of the title compound, showing the atom labelling scheme and 30% probability displacement ellipsoids.

## supplementary crystallographic information

### Comment

As part of on-going investigations of S-DABO analogues, *e.g*. 2-alkylsulfanyl-6-benzyl-3,4-dihydropyrimidin-4(3*H*) -one derivatives, which comprise a potent family of non-nucleoside reverse transcriptase inhibitors (NNRTI's), the title compound was synthesized as a novel inhibitor which shows remarkable anti-HIV-1 activity (He *et al.*, 2011).

The molecular structure is shown in Fig. 1. The cyclohexane ring adopts a chair conformation. The C12—C15—C16 angle is 113.41 (13)°, which is in the range considered optimal for maximum activity of NNRTI's, *viz*. 110–115° (Ettorre *et al.*, 1996).

A comparison of the molecular structure of the title compound with some reported S-DABO's show that their spatial arrangement are similar (Ettorre *et al.*, 1998; Rao *et al.*, 2007; Zhang *et al.*, 2008). Although these molecules assume similar conformations, they show differences in their activities. Thus, further structural investigations are needed in order to establish a structure-activity relationship.

In the crystal, molecules are connected into centrosymmetric dimers via N—H···O hydrogen bonds, Table 1.

### Experimental

With 2-cyclohexylacetonitrile as the starting material, the title compound was synthesized according to the procedure of He *et al.* (2011). Single crystals of the title compound were obtained from the slow evaporation at room temperature of its ethyl acetate/petroleum ether solution.

### Refinement

Methyl-H atoms were placed in calculated positions with C—H = 0.96 Å and the torsion angles were refined to fit the electron density; *U*_iso_(H) = 1.5*U*_eq_(C). Other H atoms were placed in calculated positions with N—H = 0.86 Å and C—H = 0.93–0.98 Å, and with *U*_iso_(H) = 1.2*U*_eq_(C, N).

### Crystal data

**Table:**

C_21_H_26_N_2_O_2_S	*Z* = 2
*M**_r_* = 370.50	*F*(000) = 396
Triclinic, *P*1	*D*_x_ = 1.240 Mg m^−^^3^
Hall symbol: -P 1	Mo *K*α radiation, λ = 0.71073 Å
*a* = 7.516 (5) Å	Cell parameters from 2756 reflections
*b* = 10.960 (5) Å	θ = 2.4–27.3°
*c* = 12.490 (5) Å	µ = 0.18 mm^−^^1^
α = 84.082 (5)°	*T* = 293 K
β = 78.925 (5)°	Block, colourless
γ = 80.267 (5)°	0.46 × 0.23 × 0.17 mm
*V* = 992.5 (9) Å^3^	

### Data collection

**Table:**

Bruker SMART CCD area-detector diffractometer	4158 independent reflections
Radiation source: fine-focus sealed tube	3203 reflections with *I* > 2σ(*I*)
graphite	*R*_int_ = 0.019
φ and ω scans	θ_max_ = 28.1°, θ_min_ = 1.7°
Absorption correction: multi-scan (*SADABS*; Bruker, 2004)	*h* = −9→9
*T*_min_ = 0.944, *T*_max_ = 0.975	*k* = −14→14
7097 measured reflections	*l* = −16→16

### Refinement

**Table:**

Refinement on *F*^2^	Secondary atom site location: difference Fourier map
Least-squares matrix: full	Hydrogen site location: inferred from neighbouring sites
*R*[*F*^2^ > 2σ(*F*^2^)] = 0.040	H-atom parameters constrained
*wR*(*F*^2^) = 0.129	*w* = 1/[σ^2^(*F*_o_^2^) + (0.0735*P*)^2^ + 0.0929*P*] where *P* = (*F*_o_^2^ + 2*F*_c_^2^)/3
*S* = 1.03	(Δ/σ)_max_ < 0.001
4158 reflections	Δρ_max_ = 0.18 e Å^−^^3^
237 parameters	Δρ_min_ = −0.18 e Å^−^^3^
0 restraints	Extinction correction: *SHELXL97* (Sheldrick, 2008), Fc^*^=kFc[1+0.001xFc^2^λ^3^/sin(2θ)]^-1/4^
Primary atom site location: structure-invariant direct methods	Extinction coefficient: 0.042 (4)

### Special details

**Table:**

Geometry. All e.s.d.'s (except the e.s.d. in the dihedral angle between two l.s. planes)are estimated using the full covariance matrix. The cell e.s.d.'s are takeninto account individually in the estimation of e.s.d.'s in distances, anglesand torsion angles; correlations between e.s.d.'s in cell parameters are onlyused when they are defined by crystal symmetry. An approximate (isotropic)treatment of cell e.s.d.'s is used for estimating e.s.d.'s involving l.s. planes.
Refinement. Refinement of *F*^2^ against ALL reflections. The weighted *R*-factor *wR* and goodness of fit *S* are based on *F*^2^, conventional *R*-factors *R* are based on *F*, with *F* set to zero for negative *F*^2^. The threshold expression of *F*^2^ > σ(*F*^2^) is used only for calculating *R*-factors(gt) *etc*, and is not relevant to the choice of reflections for refinement. *R*-factors based on *F*^2^ are statistically about twice as large as those based on *F*, and *R*-factors based on ALL data will be even larger.

### Fractional atomic coordinates and isotropic or equivalent isotropic displacement parameters (Å^2^)

**Table:**

	*x*	*y*	*z*	*U*_iso_*/*U*_eq_	
C1	0.0651 (2)	0.34455 (19)	0.37479 (16)	0.0637 (5)	
H1	0.0740	0.2786	0.4276	0.076*	
C2	−0.0742 (3)	0.3611 (2)	0.31452 (19)	0.0768 (6)	
H2	−0.1563	0.3044	0.3255	0.092*	
C3	−0.0931 (3)	0.4597 (2)	0.23879 (17)	0.0735 (6)	
H3	−0.1883	0.4709	0.1992	0.088*	
C4	0.0298 (3)	0.5416 (2)	0.22211 (17)	0.0707 (6)	
H4	0.0174	0.6092	0.1711	0.085*	
C5	0.1713 (3)	0.52527 (17)	0.27987 (16)	0.0622 (5)	
H5	0.2543	0.5815	0.2671	0.075*	
C6	0.1917 (2)	0.42627 (15)	0.35661 (13)	0.0483 (4)	
C7	0.3555 (2)	0.40826 (14)	0.41151 (14)	0.0490 (4)	
C8	0.3441 (2)	0.33926 (16)	0.52377 (14)	0.0518 (4)	
H8A	0.2644	0.2772	0.5284	0.062*	
H8B	0.2876	0.3975	0.5784	0.062*	
C9	0.6130 (2)	0.14968 (13)	0.46180 (12)	0.0409 (3)	
C10	0.8364 (2)	−0.02021 (14)	0.39454 (12)	0.0451 (4)	
C11	0.7259 (2)	−0.02517 (14)	0.31332 (12)	0.0436 (4)	
C12	0.5655 (2)	0.05556 (14)	0.31667 (12)	0.0426 (4)	
C13	0.8014 (3)	−0.12053 (17)	0.23150 (14)	0.0548 (4)	
H13A	0.7058	−0.1299	0.1921	0.066*	
H13B	0.8355	−0.1997	0.2704	0.066*	
C14	0.9669 (3)	−0.0886 (2)	0.14963 (16)	0.0727 (6)	
H14A	0.9352	−0.0095	0.1116	0.109*	
H14B	1.0048	−0.1512	0.0980	0.109*	
H14C	1.0655	−0.0849	0.1874	0.109*	
C15	0.4356 (2)	0.06173 (16)	0.23778 (14)	0.0529 (4)	
H15A	0.3134	0.0568	0.2789	0.063*	
H15B	0.4723	−0.0096	0.1942	0.063*	
C16	0.4293 (2)	0.18009 (16)	0.16104 (13)	0.0504 (4)	
H16	0.3873	0.2507	0.2065	0.060*	
C17	0.6149 (3)	0.19746 (19)	0.09419 (16)	0.0658 (5)	
H17A	0.6629	0.1263	0.0514	0.079*	
H17B	0.6992	0.2017	0.1431	0.079*	
C18	0.6038 (4)	0.3151 (2)	0.0178 (2)	0.0849 (7)	
H18A	0.5672	0.3869	0.0608	0.102*	
H18B	0.7238	0.3211	−0.0259	0.102*	
C19	0.4667 (4)	0.3152 (2)	−0.05721 (19)	0.0914 (8)	
H19A	0.4570	0.3930	−0.1019	0.110*	
H19B	0.5099	0.2485	−0.1057	0.110*	
C20	0.2831 (4)	0.2987 (2)	0.00690 (19)	0.0825 (7)	
H20A	0.2010	0.2931	−0.0430	0.099*	
H20B	0.2335	0.3709	0.0481	0.099*	
C21	0.2904 (3)	0.1827 (2)	0.08558 (17)	0.0692 (6)	
H21A	0.3233	0.1098	0.0439	0.083*	
H21B	0.1697	0.1797	0.1296	0.083*	
N2	0.77310 (18)	0.07257 (11)	0.46550 (10)	0.0446 (3)	
H2A	0.8372	0.0817	0.5134	0.054*	
N1	0.50716 (18)	0.14361 (12)	0.39265 (10)	0.0448 (3)	
O1	0.49051 (17)	0.45336 (12)	0.36902 (12)	0.0662 (4)	
O2	0.98026 (18)	−0.09226 (11)	0.40372 (10)	0.0621 (4)	
S1	0.56039 (6)	0.26398 (4)	0.55656 (3)	0.05176 (16)	

### Atomic displacement parameters (Å^2^)

**Table:**

	*U*^11^	*U*^22^	*U*^33^	*U*^12^	*U*^13^	*U*^23^
C1	0.0452 (10)	0.0686 (12)	0.0717 (12)	−0.0097 (9)	−0.0091 (9)	0.0197 (9)
C2	0.0499 (11)	0.0943 (16)	0.0845 (14)	−0.0205 (11)	−0.0142 (10)	0.0214 (12)
C3	0.0470 (11)	0.0964 (16)	0.0684 (12)	0.0058 (11)	−0.0100 (9)	0.0057 (11)
C4	0.0724 (14)	0.0637 (12)	0.0646 (12)	0.0105 (11)	−0.0110 (10)	0.0120 (9)
C5	0.0633 (12)	0.0491 (10)	0.0674 (11)	−0.0028 (9)	−0.0045 (9)	0.0042 (8)
C6	0.0420 (9)	0.0427 (8)	0.0527 (9)	0.0021 (7)	0.0018 (7)	−0.0016 (7)
C7	0.0457 (9)	0.0372 (8)	0.0592 (10)	−0.0009 (7)	−0.0011 (7)	−0.0055 (7)
C8	0.0487 (10)	0.0530 (9)	0.0509 (9)	−0.0024 (8)	−0.0029 (7)	−0.0107 (7)
C9	0.0456 (9)	0.0369 (7)	0.0395 (7)	−0.0058 (6)	−0.0076 (6)	−0.0002 (6)
C10	0.0532 (10)	0.0387 (8)	0.0439 (8)	−0.0027 (7)	−0.0134 (7)	−0.0029 (6)
C11	0.0516 (9)	0.0411 (8)	0.0393 (8)	−0.0107 (7)	−0.0093 (6)	−0.0013 (6)
C12	0.0472 (9)	0.0437 (8)	0.0390 (7)	−0.0127 (7)	−0.0099 (6)	0.0015 (6)
C13	0.0617 (11)	0.0549 (10)	0.0488 (9)	−0.0067 (8)	−0.0091 (8)	−0.0131 (7)
C14	0.0771 (14)	0.0703 (13)	0.0607 (11)	−0.0026 (11)	0.0067 (10)	−0.0082 (9)
C15	0.0548 (10)	0.0564 (10)	0.0533 (9)	−0.0147 (8)	−0.0184 (8)	−0.0041 (8)
C16	0.0554 (10)	0.0515 (9)	0.0475 (9)	−0.0019 (8)	−0.0197 (7)	−0.0093 (7)
C17	0.0657 (12)	0.0642 (12)	0.0646 (11)	−0.0061 (10)	−0.0143 (9)	0.0076 (9)
C18	0.0946 (18)	0.0703 (14)	0.0837 (15)	−0.0142 (13)	−0.0134 (13)	0.0207 (12)
C19	0.134 (2)	0.0711 (15)	0.0622 (13)	0.0098 (15)	−0.0294 (14)	0.0062 (11)
C20	0.1008 (19)	0.0758 (14)	0.0750 (14)	0.0137 (13)	−0.0477 (14)	−0.0086 (11)
C21	0.0726 (14)	0.0746 (13)	0.0682 (12)	−0.0044 (11)	−0.0350 (10)	−0.0099 (10)
N2	0.0524 (8)	0.0418 (7)	0.0421 (7)	−0.0032 (6)	−0.0171 (6)	−0.0049 (5)
N1	0.0461 (8)	0.0456 (7)	0.0443 (7)	−0.0062 (6)	−0.0122 (6)	−0.0038 (5)
O1	0.0512 (8)	0.0637 (8)	0.0807 (9)	−0.0157 (6)	−0.0052 (6)	0.0069 (7)
O2	0.0699 (9)	0.0519 (7)	0.0667 (8)	0.0136 (6)	−0.0314 (6)	−0.0164 (6)
S1	0.0566 (3)	0.0501 (3)	0.0499 (3)	−0.00196 (19)	−0.01321 (19)	−0.01319 (18)

### Geometric parameters (Å, °)

**Table:**

C1—C2	1.380 (3)	C13—C14	1.516 (3)
C1—C6	1.386 (2)	C13—H13A	0.9700
C1—H1	0.9300	C13—H13B	0.9700
C2—C3	1.368 (3)	C14—H14A	0.9600
C2—H2	0.9300	C14—H14B	0.9600
C3—C4	1.368 (3)	C14—H14C	0.9600
C3—H3	0.9300	C15—C16	1.531 (2)
C4—C5	1.374 (3)	C15—H15A	0.9700
C4—H4	0.9300	C15—H15B	0.9700
C5—C6	1.380 (3)	C16—C17	1.513 (3)
C5—H5	0.9300	C16—C21	1.530 (2)
C6—C7	1.497 (3)	C16—H16	0.9800
C7—O1	1.210 (2)	C17—C18	1.523 (3)
C7—C8	1.517 (2)	C17—H17A	0.9700
C8—S1	1.795 (2)	C17—H17B	0.9700
C8—H8A	0.9700	C18—C19	1.520 (3)
C8—H8B	0.9700	C18—H18A	0.9700
C9—N1	1.295 (2)	C18—H18B	0.9700
C9—N2	1.354 (2)	C19—C20	1.486 (4)
C9—S1	1.7563 (16)	C19—H19A	0.9700
C10—O2	1.243 (2)	C19—H19B	0.9700
C10—N2	1.381 (2)	C20—C21	1.524 (3)
C10—C11	1.440 (2)	C20—H20A	0.9700
C11—C12	1.366 (2)	C20—H20B	0.9700
C11—C13	1.502 (2)	C21—H21A	0.9700
C12—N1	1.382 (2)	C21—H21B	0.9700
C12—C15	1.504 (2)	N2—H2A	0.8600
			
C2—C1—C6	120.01 (18)	H14A—C14—H14C	109.5
C2—C1—H1	120.0	H14B—C14—H14C	109.5
C6—C1—H1	120.0	C12—C15—C16	113.41 (13)
C3—C2—C1	120.9 (2)	C12—C15—H15A	108.9
C3—C2—H2	119.5	C16—C15—H15A	108.9
C1—C2—H2	119.5	C12—C15—H15B	108.9
C4—C3—C2	119.1 (2)	C16—C15—H15B	108.9
C4—C3—H3	120.5	H15A—C15—H15B	107.7
C2—C3—H3	120.5	C17—C16—C21	110.13 (16)
C3—C4—C5	120.76 (19)	C17—C16—C15	113.00 (15)
C3—C4—H4	119.6	C21—C16—C15	110.86 (15)
C5—C4—H4	119.6	C17—C16—H16	107.5
C4—C5—C6	120.67 (19)	C21—C16—H16	107.5
C4—C5—H5	119.7	C15—C16—H16	107.5
C6—C5—H5	119.7	C16—C17—C18	111.75 (17)
C5—C6—C1	118.49 (18)	C16—C17—H17A	109.3
C5—C6—C7	118.30 (16)	C18—C17—H17A	109.3
C1—C6—C7	123.11 (16)	C16—C17—H17B	109.3
O1—C7—C6	120.20 (16)	C18—C17—H17B	109.3
O1—C7—C8	121.06 (16)	H17A—C17—H17B	107.9
C6—C7—C8	118.65 (14)	C19—C18—C17	111.2 (2)
C7—C8—S1	114.80 (12)	C19—C18—H18A	109.4
C7—C8—H8A	108.6	C17—C18—H18A	109.4
S1—C8—H8A	108.6	C19—C18—H18B	109.4
C7—C8—H8B	108.6	C17—C18—H18B	109.4
S1—C8—H8B	108.6	H18A—C18—H18B	108.0
H8A—C8—H8B	107.5	C20—C19—C18	111.0 (2)
N1—C9—N2	123.68 (14)	C20—C19—H19A	109.4
N1—C9—S1	121.55 (12)	C18—C19—H19A	109.4
N2—C9—S1	114.75 (11)	C20—C19—H19B	109.4
O2—C10—N2	119.81 (14)	C18—C19—H19B	109.4
O2—C10—C11	124.65 (14)	H19A—C19—H19B	108.0
N2—C10—C11	115.54 (14)	C19—C20—C21	112.29 (19)
C12—C11—C10	118.33 (14)	C19—C20—H20A	109.1
C12—C11—C13	126.18 (15)	C21—C20—H20A	109.1
C10—C11—C13	115.48 (15)	C19—C20—H20B	109.1
C11—C12—N1	122.99 (14)	C21—C20—H20B	109.1
C11—C12—C15	124.59 (14)	H20A—C20—H20B	107.9
N1—C12—C15	112.40 (14)	C20—C21—C16	112.09 (17)
C11—C13—C14	113.75 (15)	C20—C21—H21A	109.2
C11—C13—H13A	108.8	C16—C21—H21A	109.2
C14—C13—H13A	108.8	C20—C21—H21B	109.2
C11—C13—H13B	108.8	C16—C21—H21B	109.2
C14—C13—H13B	108.8	H21A—C21—H21B	107.9
H13A—C13—H13B	107.7	C9—N2—C10	121.91 (13)
C13—C14—H14A	109.5	C9—N2—H2A	119.0
C13—C14—H14B	109.5	C10—N2—H2A	119.0
H14A—C14—H14B	109.5	C9—N1—C12	117.43 (14)
C13—C14—H14C	109.5	C9—S1—C8	99.15 (8)
			
C6—C1—C2—C3	−2.0 (4)	C11—C12—C15—C16	−110.85 (18)
C1—C2—C3—C4	0.9 (4)	N1—C12—C15—C16	67.63 (19)
C2—C3—C4—C5	0.4 (3)	C12—C15—C16—C17	57.7 (2)
C3—C4—C5—C6	−0.5 (3)	C12—C15—C16—C21	−178.14 (15)
C4—C5—C6—C1	−0.6 (3)	C21—C16—C17—C18	54.8 (2)
C4—C5—C6—C7	175.91 (16)	C15—C16—C17—C18	179.37 (17)
C2—C1—C6—C5	1.9 (3)	C16—C17—C18—C19	−56.5 (3)
C2—C1—C6—C7	−174.50 (18)	C17—C18—C19—C20	55.8 (3)
C5—C6—C7—O1	−21.6 (2)	C18—C19—C20—C21	−54.8 (3)
C1—C6—C7—O1	154.81 (18)	C19—C20—C21—C16	54.2 (2)
C5—C6—C7—C8	154.89 (16)	C17—C16—C21—C20	−53.3 (2)
C1—C6—C7—C8	−28.7 (2)	C15—C16—C21—C20	−179.13 (18)
O1—C7—C8—S1	−29.6 (2)	N1—C9—N2—C10	−0.1 (2)
C6—C7—C8—S1	153.97 (12)	S1—C9—N2—C10	−178.32 (11)
O2—C10—C11—C12	176.35 (16)	O2—C10—N2—C9	−176.98 (14)
N2—C10—C11—C12	−3.6 (2)	C11—C10—N2—C9	3.0 (2)
O2—C10—C11—C13	−4.0 (2)	N2—C9—N1—C12	−2.1 (2)
N2—C10—C11—C13	176.04 (13)	S1—C9—N1—C12	175.98 (10)
C10—C11—C12—N1	1.7 (2)	C11—C12—N1—C9	1.3 (2)
C13—C11—C12—N1	−177.99 (14)	C15—C12—N1—C9	−177.25 (13)
C10—C11—C12—C15	179.99 (14)	N1—C9—S1—C8	2.47 (15)
C13—C11—C12—C15	0.3 (2)	N2—C9—S1—C8	−179.29 (11)
C12—C11—C13—C14	107.9 (2)	C7—C8—S1—C9	−67.38 (14)
C10—C11—C13—C14	−71.8 (2)		

### Hydrogen-bond geometry (Å, °)

**Table:**

*D*—H···*A*	*D*—H	H···*A*	*D*···*A*	*D*—H···*A*
N2—H2A···O2^i^	0.86	1.90	2.743 (2)	168

Symmetry codes: (i) −*x*+2, −*y*, −*z*+1.
